# Comorbidity burden and survival in patients with idiopathic pulmonary fibrosis: the EMPIRE registry study

**DOI:** 10.1186/s12931-022-02033-6

**Published:** 2022-05-27

**Authors:** Dragana M. Jovanovic, Martina Šterclová, Nesrin Mogulkoc, Katarzyna Lewandowska, Veronika Müller, Marta Hájková, Michael Studnicka, Jasna Tekavec-Trkanjec, Simona Littnerová, Martina Vašáková, the EMPIRE registry investigators, Stefan Zembacher, Michael Studnicka, David Lang, Bernd Lamprecht, Natalia Stoeva, Suzana Mladinov, Dino Ilak, Mirna Vergles, Neven Tudorić, Jasna Tekavec-Trkanjec, Martina Vašáková, Martina Šterclová, Jiří, Ladislav Lacina, Pavlína Lisá, Radka Bittenglová, Vladimír Bartoš, Renata, Vladimíra Lošťáková, Monika Žurková, Jana Pšíkalová, Ilona Binková, Martina Doubková, Jan Kervitzer, Tomáš Snížek, Pavel Reiterer, Hana Šuldová, Martina Plačková, Richard Tyl, Vladimír Řihák, Ladislav Dušek, Karel Hejduk, Jakub Gregor, Ondřej Májek, Simona Littnerová, Michal Svoboda, Miklós Zsiray, Veronika Müller, Anikó Bohács, Maria Szilasi, Zsuzsanna Szalai, Zoltán Balikó, Attila Somfay, Imre Lajkó, Mordechai Kramer, Yochai Adir, Biserka Jovkovska Kjaeva, Ewa Jassem, Alicja Sieminska, Amelia Szymanowska-Narloch, Adam Barczyk, Krzysztof Sladek, Aleksander Kania, Lukasz Kasper, Tomasz Stachura, Paulina Jurek, Sebastian Majewski, Agata Nowicka, Lukasz Borucki, Katarzyna Lewandowska, Małgorzata Sobiecka, Beate Zolnowska, Pawel Sliwinski, Damian Korzybski, Magdalena Martusewicz-Boros, Elzbieta Wiatr, Marta Maskey-Warzęchowska, Katarzyna Górska, Małgorzata Barnaś, Violeta Vučinić-Mihailović, Branislava Milenković, Milica Kontić, Natalija Samardžić, Marina Roksandić, Dragana Jovanovic, Tatjana Pejcic, Desa Nastasijevic Boravac, Emilija Videnovic, Tatjana Radjenovic Petkovic, Ivanka Djordejevic, Svetlana Kašiković Lečić, Vesna Kuruc, Živka Eri, Milana Panjković, Aleksandra Lovrenski, Darinka Kukavica, Ana Jakić, Slavica Mojsilovic, Marta Hájková, Štefan Laššán, Štefan Tóth, Ján Plutinský, Róbert Vyšehradský, Bohumil Matula, Róbert Slivka, Imrich Jonner, Nesrin Mogulkoc

**Affiliations:** 1 Internal Medicine, Clinic Akta Medica, Belgrade, Serbia; 2 Department of Respiratory Medicine, First Faculty of Medicine, Charles University, Thomayer Hospital, Prague, Czech Republic; 3 Department of Pulmonary Medicine, Ege University Medical School, Izmir, Turkey; 4 Department of Pulmonary Diseases, National Research Institute of Tuberculosis and Lung Diseases, Warsaw, Poland; 5 Department of Pulmonology, Semmelweis University, Budapest, Hungary; 6 Clinic of Pneumology and Phthisiology, University Hospital Bratislava, Bratislava, Slovakia; 7 Department of Pneumology, Paracelsus Medical University, Salzburg, Austria; 8 Pulmonary Department, University Hospital Dubrava, Zagreb, Croatia; 9 Institute of Biostatistics and Analyses, Faculty of Medicine, Masaryk University, Brno, Czechia

**Keywords:** EMPIRE, Mortality, Idiopathic pulmonary fibrosis, Registry

## Abstract

**Background:**

Patients with idiopathic pulmonary fibrosis (IPF) frequently have multiple comorbidities, which may influence survival but go under-recognised in clinical practice. We therefore report comorbidity, antifibrotic treatment use and survival of patients with IPF observed in the multi-national EMPIRE registry.

**Methods:**

For this prospective IPF cohort, demographics, comorbidities, survival and causes of death were analysed. Comorbidities were noted by the treating physician based on the patient’s past medical history or as reported during follow-up. Comorbidities were defined as prevalent when noted at enrolment, or as incident when recorded during follow-up. Survival was analysed by Kaplan–Meier estimates, log-rank test, and Cox proportional hazards models. Hazard ratios (HR) were adjusted for gender, age, smoking status and FVC at enrolment.

**Results:**

A population of 3,580 patients with IPF from 11 Central and Eastern European countries was followed every 6 months for up to 6 years. At enrolment, 91.3% of patients reported at least one comorbidity, whereas more than one-third (37.8%) reported four or more comorbidities. Five-year survival was 53.7% in patients with no prevalent comorbidities, whereas it was 48.4%, 47.0%, 43.8% and 41.1% in patients with 1, 2, 3 and ≥ 4 comorbidities, respectively. The presence of multiple comorbidities at enrolment was associated with significantly worse survival (log-rank test *P* = 0.007). Adjusted HRs indicate that risk of death was increased by 44% in patients with IPF reporting ≥ 4 comorbidities at baseline compared with no comorbidity (*P* = 0.021). The relationship between number of comorbidities and decreased survival was also seen in patients receiving antifibrotic treatment (63% of all patients; log-rank test *P* < 0.001). Comorbidity as cause of death was identified in at least 26.1% of deaths.

**Conclusions:**

The majority of patients with IPF demonstrate comorbidities, and many have comorbidity-related deaths. Increasing numbers of comorbidities are associated with worse survival; and this pattern is also present in patients receiving antifibrotic therapy.

**Supplementary Information:**

The online version contains supplementary material available at 10.1186/s12931-022-02033-6.

## Introduction

Idiopathic pulmonary fibrosis (IPF) is a devastating interstitial lung disease primarily affecting older adults, associated with progressive loss of lung function [1]. Prevalence estimates vary widely, ranging from 1.25 to 63 IPF cases per 100,000 persons, depending on definitions and study methodology [2–5]. Prognosis for IPF is poor and mortality rates are high, with a reported survival of 3–5 years after diagnosis [6, 7].

In patients with IPF, symptomatic disease is very frequent and associated with increased healthcare costs [8]. In addition to a high symptom burden, many patients have pulmonary or extrapulmonary comorbidities that can negatively impact on quality of life and survival [9, 10]. Causes of comorbidities include shared underlying risk factors such as age and smoking; IPF itself leading to hypoxemia and pulmonary hypertension; medications used for IPF treatment, such as diabetes mellitus following treatment with steroids; and a combination of these mechanisms [11, 12]. Recent data indicate that some of these associations are driven by genetic variations [13]. Coronary heart disease [14], diabetes mellitus [15], pulmonary hypertension [16, 17], chronic obstructive pulmonary disease (COPD) [12, 18], obstructive sleep apnoea (OSA) [19], gastroesophageal reflux [20, 21], and lung cancer [22] are all frequently associated with IPF. Given the effect of comorbidities on patients’ quality of life [10] and survival [9], comprehensive assessment of patients with IPF should include early recognition and appropriate management of comorbidities [23].

Antifibrotic treatment of IPF with nintedanib or pirfenidone can slow lung function decline, decrease the risk for acute exacerbation, and reduce mortality [24–27]. However, comorbidities, especially if left unrecognised and untreated, can have a negative impact on survival in patients with IPF and thus might reduce the positive effect of antifibrotic treatment [9, 23].

The reported prevalence of comorbidities in IPF varies widely, depending on the type of study or methodology involved (i.e., whether data are derived from clinical trials, patient registries or observational data), the population studied, and the diagnostic criteria applied. However, when data for comorbidities in IPF are compared from a single type of study, such as IPF patient registries, the pattern is more consistent [28].

We aimed to estimate the prevalence of comorbidities at the time of enrolment in the European MultiPartner IPF REgistry (EMPIRE) as well as the incidence of comorbidities during follow-up, and to analyse the impact on survival. For a well-defined IPF cohort, we included 3,580 patients from Central and Eastern Europe, with the EMPIRE registry currently the largest database of this kind [29].

In EMPIRE, patients were seen at least every 6 months and the presence of comorbidities assessed using a pre-defined, standardised, web-based data entry tool. We describe and analyse comorbidities in two ways. Firstly, we number comorbidities present at enrolment (prevalent), and secondly, we report the total number of comorbidities recorded at enrolment and up to the end of follow-up.

## Methods

EMPIRE is a non-interventional, multinational registry of patients with IPF that was established in September 2014 (http://empire.registry.cz/index-en.php). The registry currently collects data from 11 Central and Eastern European countries: Austria, Bulgaria, Croatia, Czech Republic, Hungary, Israel, Macedonia, Poland, Serbia, Slovakia, and Turkey [29]. Patient data were entered into the registry database by the treating physician. When a patient completed their follow-up visit, clinical data were recorded using an online database based on the TrialDB system [28]. All data transfers were encrypted. Data validation took place both during data input (validation and testing for internal consistency between variables) and during data processing (identification and exclusion of missing, outlying, and otherwise implausible observations).

EMPIRE is conducted in accordance with the Declaration of Helsinki. The study protocol and its amendments were approved by the Ethics Committee of each participating institution. All records in the EMPIRE registry are fully anonymised and de-identified.

Data for all patients registered in EMPIRE between 5 December 2014 and 14 May 2020 were extracted for analysis. To be eligible for inclusion in this analysis, patients had to have a diagnosis of IPF defined according to 2011 European Respiratory Society/American Thoracic Society/Japanese Respiratory Society/Latin American Thoracic Society criteria [30, 31]. Final IPF diagnosis was always determined by a local multidisciplinary team (MDT). The date of IPF diagnosis was the date recorded by the treating physician. Patients were excluded if the date of diagnosis was unknown or if IPF diagnosis was changed during follow-up.

Multidisciplinary team diagnosis was reached according to the operational principles of study centres and strictly followed published guidelines. Date of IPF diagnosis, demographic characteristics, lung function, comorbidities, antifibrotic treatment, survival status, and cause of death were captured for analysis. Comorbidities were noted by the treating physician based on a patient’s medical history or as reported during follow-up.

Comorbidities present at the time of the first visit to EMPIRE (enrolment visit) were recorded as prevalent comorbidities, and comorbidities noted at the time of enrolment and during follow-up were recorded as the cumulative number of comorbidities. Prevalent comorbidities and the cumulative number of comorbidities were grouped according to the organ/ system involved (cardiovascular; gastrointestinal, metabolic; urogenital; pulmonary; blood and immunity; other) and classified according to specific disease (e.g., arterial hypertension, coronary heart disease, pulmonary hypertension). For survival analyses, patients were categorised according to the number of comorbidities (0, 1, 2, 3, and ≥ 4) as present at enrolment (prevalent), and as from enrolment to the end of follow-up (cumulative number).

Treatment of IPF with antifibrotics (pirfenidone or nintedanib) was recorded by the treating physician. Cause of death was recorded by the treating physician and then categorised centrally.

### Statistical methods

Characteristics of patients from the time of enrolment in the registry and up to the end of follow-up are reported. Continuous variables are presented as median (5th and 95th percentile), and categorical outcomes are described by absolute and relative frequencies. The Kruskal–Wallis test was used for continuous data, and the maximum-likelihood chi-squared test was used for categorical data. For survival analyses, survival time was defined as the time between date of enrolment and date of death, loss to follow-up, or the end of follow-up defined by data analysis (i.e., 14 May 2020), whichever was earliest.

To analyse survival, Kaplan–Meier graphs were derived, and log-rank tests used to assess the difference between survival curves according to the number of comorbidities. For multiple comparisons, a Bonferroni correction was used to assess the difference between groups of patients. The association between the number of comorbidities (0 vs 1, 0 vs 2, 0 vs 3, and 0 vs ≥ 4) and survival was further described using Cox proportional hazards models; both unadjusted and adjusted (for gender, age, smoking status and forced vital capacity [FVC] % predicted at enrolment) estimates are presented. Statistical significance was set at *P* < 0.05.

## Results

### Study population

As of 14 May 2020, data from the EMPIRE registry were available for 3580 patients with an MDT-diagnosis of IPF; of those, 3286 (91.8%) had a high-resolution computed tomography scan, and 798 (22.3%) underwent lung biopsy (Table 1).

**Table 1:** Baseline characteristics of patients with IPF according to the number of comorbidities at enrolment

	Total (N = 3580)	Number of comorbidities at enrolment					*P*-value ^a^
		0 (n = 310)	1 (n = 581)	2 (n = 670)	3 (n = 667)	≥ 4 (n = 1352)	
Sex, n (%)							
Female	1050 (29.3)	93 (30.0)	168 (28.9)	226 (33.7)	203 (30.4)	360 (26.6)	0.022
Male	2530 (70.7)	217 (70.0)	413 (71.1)	444 (66.3)	464 (69.6)	992 (73.4)	
Age at enrolment, years (median, 5th; 95th percentile)	69 (53;82)	64 (45;78)	67 (51;82)	69 (53;82)	69 (55;81)	71 (58;83)	< 0.001
Time from diagnosis to enrolment, months (median, 5th; 95th percentile)	0.46 (0.0;63.3)	0.49 (0.00;73.67)	0.03 (0.00;46.43)	0.56 (0.00;71.84)	0.69 (0.00;66.85)	0.56 (0.00;61.90)	< 0.001
Median BMI, kg/m^2^ (5; 95 percentile)	28.0 (21.5;35.9)	26.6 (21.3;32.8)	27.5 (21.0;35.4)	27.8 (21.4;35.9)	28.4 (21.5;36.0)	28.4 (21.9;36.5)	< 0.001
Smoking, n (%)							
Never-smoker	1353 (38.0)	137 (45.5)	254 (44.3)	261 (39.0)	248 (37.3)	453 (33.5)	< 0.001
Current smoker	169 (4.7)	18 (6.0)	31 (5.4)	31 (4.6)	34 (5.1)	55 (4.1)	
Ex-smoker	2039 (57.3)	146 (48.5)	289 (50.3)	377 (56.4)	383 (57.6)	844 (62.4)	
Dyspnoea (NYHA categories, %)							
I	361 (10.7)	51 (19.0)	70 (13.2)	70 (11.1)	75 (11.8)	95 (7.2)	< 0.001
II	1762 (52.1)	152 (56.5)	303 (57.0)	347 (54.8)	321 (50.5)	639 (48.7)	
III	1183 (35.0)	63 (23.4)	151 (28.4)	194 (30.6)	226 (35.5)	549 (41.8)	
IV	77 (2.3)	3 (1.1)	8 (1.5)	22 (3.5)	14 (2.2)	30 (2.3)	
HRCT, n (%)							
UIP	2297 (64.2)	195 (62.9)	374 (64.4)	445 (66.4)	426 (63.9)	857 (63.4)	0.864
Possible UIP	862 (24.1)	75 (24.2)	148 (25.5)	155 (23.1)	162 (24.3)	322 (23.8)	
Inconsistent with UIP	127 (3.5)	10 (3.2)	21 (3.6)	22 (3.3)	24 (3.6)	50 (3.7)	
Not performed	294 (8.2)	30 (9.7)	38 (6.5)	48 (7.2)	55 (8.2)	123 (9.1)	
Histopathology, n (%)							
UIP	418 (11.7)	57 (18.4)	74 (12.7)	90 (13.4)	65 (9.7)	132 (9.8)	0.009
Probable UIP	143 (4.0)	16 (5.2)	25 (4.3)	31 (4.6)	25 (3.7)	46 (3.4)	
Possible UIP	119 (3.3)	10 (3.2)	18 (3.1)	24 (3.6)	20 (3.0)	47 (3.5)	
Not UIP	118 (3.3)	14 (4.5)	24 (4.1)	19 (2.8)	22 (3.3)	39 (2.9)	
Not performed	2782 (77.7)	213 (68.7)	440 (75.7)	506 (75.5)	535 (80.2)	1088 (80.5)	
Antifibrotic treatment, n (%)							
Pirfenidone	1098 (30.6)	72 (23.2)	150 (25.8)	183 (27.3)	220 (33.0)	473 (35.0)	< 0.001
Nintedanib	917 (25.6)	99 (31.9)	169 (29.1)	187 (27.9)	153 (22.9)	309 (22.9)	
Switch pirfenidone to nintedanib	165 (4.6)	11 (3.5)	20 (3.4)	40 (6.0)	36 (5.4)	58 (4.3)	
Switch nintedanib to pirfenidone	77 (2.1)	7 (2.3)	12 (2.1)	12 (1.8)	10 (1.5)	36 (2.7)	
No antifibrotic treatment	1323 (37.0)	121 (39.0)	230 (39.6)	248 (37.0)	248 (37.2)	476 (35.2)	
Lung function parameters, median (5; 95 percentile)							
FVC, % predicted	77 (45;114)	73 (43;112)	77 (49;116)	79 (47;117)	78 (45;112)	75 (44;112)	0.000
FEV_1_, % predicted	81 (48;114)	79 (47;113)	83 (53;122)	82 (49;116)	81 (49;112)	79 (46;110)	0.000
TLC, % predicted	47.4 (22.2;81.5)	48.0 (23.0;84.0)	48.4 (23.4;82.5)	49.4 (22.7;84.8)	48.2 (25.7;80.6)	45.1 (21.1;77.9)	0.001
6MWD, m	391 (165;558)	450 (240;605)	420 (165;572)	393 (170;543)	398 (150;568)	370 (150;525)	0.000

*6MWD* 6-min walking distance, *BMI* body mass index, *EMPIRE* European Multipartner IPF REgistry, *FEV*_*1*_ forced expiratory volume in 1 s, *FVC* forced vital capacity, *HRCT* high-resolution computed tomography, *IPF* idiopathic pulmonary fibrosis, *ML* maximum likelihood, *NYHA* New York Heart Association functional classification, *TLC* total lung capacity, *UIP* usual interstitial pneumonia

^a^Statistical significance tested by Kruskal–Wallis test for continuous parameters and by ML chi-square for categorical variables

Data originated from 11 countries, with patients from the Czech Republic accounting for one-third (*n* = 1174; 32.8%) of the analysis population. At the time of analysis, 949/3580 (26.5%) patients had died and 451 (12.6%) had observations censored before the end of follow-up; of these, 331 were lost to follow-up; 73 underwent lung transplantation; and 47 were censored for other, unspecified reasons. Patient characteristics at enrolment are presented in Table 1. Median (5th–95th percentile) age at registry enrolment was 69 (53–82) years, and more than two-thirds of patients (70.7%) were male.

### Comorbidities at enrolment and during follow-up

At the time of enrolment, most patients (91.3%) had at least one comorbidity, 56.4% had at least three, and more than one-third (37.8%) had at least four comorbidities. The number of comorbidities increased with age, as expected. Median follow-up time was 13.8 months (5th–95th percentile 0.0–58.3 months).

Most patients (73.9%) had a cardiovascular type of comorbidity recorded either at baseline or during follow-up. The most common disease-specific comorbidities were arterial hypertension (53.0%), diabetes mellitus (24.0%), hyperlipidaemia (23.5%), coronary heart disease (23.3%), and gastroesophageal reflux (21.1%) (Table 2). Less common, but still affecting ≥ 5– < 10% of patients, were comorbidities including cancer other than lung cancer, depression, thyroid disease, respiratory infection, obesity, COPD, embolism and heart attack (Table 2).

**Table 2:** Comorbidities in patients with IPF from the EMPIRE Registry

	Prevalence		Incidence	
Comorbidity	Patients, n (%) (N = 3580)	Range across participating countries, %	Patients, n (%) (N = 3580)	Range across participating countries, %
Cardiovascular	2646 (73.9)	66.6–82.4	2377 (66.4)	53.3–82.4
Arterial hypertension	1896 (53.0)	42.5–64.7	1645 (45.9)	33.2–64.7
Coronary heart disease	834 (23.3)	8.5–35.8	691 (19.3)	7.8–33.3
Pulmonary hypertension	418 (11.7)	4.4–40.4	362 (10.1)	4.0–40.4
Arrhythmias	378 (10.6)	3.9–23.5	344 (9.6)	2.6–23.5
Heart attack	213 (5.9)	0.0–11.8	190 (5.3)	0.0–11.4
Embolism	199 (5.6)	0.0–10.1	177 (4.9)	0.0–8.9
Stroke	156 (4.4)	0.0–10.3	140 (3.9)	0.0–10.3
Valve disease	154 (4.3)	0.3–11.8	135 (3.8)	0.0–11.8
Ischaemic disease of the lower limbs	82 (2.3)	0.0–4.3	74 (2.1)	0.0–3.7
Cardiomyopathy	44 (1.2)	0.0–6.5	38 (1.1)	0.0–5.9
Other	380 (10.6)	3.9–16.3	317 (8.9)	3.5–15.6
Pulmonary	1396 (39.0)	5.9–68.5	1065 (29.7)	5.9–58.7
Emphysema	365 (10.2)	0.0–40.6	125 (3.5)	0.0–26.9
Tuberculosis	243 (6.8)	0.0–29.6	63 (1.8)	0.0–4.3
COPD	235 (6.6)	0.0–22.1	210 (5.9)	0.0–21.2
Respiratory infection	231 (6.5)	0.0–12.5	229 (6.4)	0.0–12.4
Respiratory insufficiency	230 (6.4)	0.0–21.2	194 (5.4)	0.0–21.2
Asthma	160 (4.5)	0.0–9.6	127 (3.5)	0.0–7.8
Lung cancer	128 (3.6)	0.9–7.4	93 (2.6)	0.9–6.7
Pneumonia	116 (3.2)	0.0–6.7	104 (2.9)	0.0–6.5
Obstructive sleep apnoea	99 (2.8)	0.0–17.1	92 (2.6)	0.0–16.7
Other	246 (6.9)	0.0–17.6	160 (4.5)	0.0–6.5
Gastrointestinal/metabolic	2119 (59.2)	28.1–92.3	1839 (51.4)	27.5–88.6
Diabetes mellitus	860 (24.0)	15.2–37.0	762 (21.3)	14.2–36.6
Hyperlipidaemia	843 (23.5)	0.0–72.8	720 (20.1)	0.0–67.9
Gastroesophageal reflux	755 (21.1)	8.5–54.8	639 (17.8)	8.4–51.0
Thyroid diseases	255 (7.1)	0.0–15.0	224 (6.3)	0.0–13.8
Obesity	208 (5.8)	0.0–34.1	144 (4.0)	0.0–30.1
Hepatopathy	125 (3.5)	0.0–10.2	109 (3.0)	0.0–8.9
Other	556 (15.5)	7.2–29.7	489 (13.7)	6.4–29.7
Urogenital	665 (18.6)	0.0–45.1	591 (16.5)	0.0–44.3
Prostatic hypertrophy	432 (12.1)	0.0–24.8	385 (10.8)	0.0–24.0
Nephropathy	129 (3.6)	0.0–15.9	115 (3.2)	0.0–15.9
Other	147 (4.1)	0.0–15.0	134 (3.7)	0.0–14.6
Blood and immunity	229 (6.4)	0.0–15.4	187 (5.2)	0.0–15.4
Haematopoietic disorders	63 (1.8)	0.0–4.5	54 (1.5)	0.0–4.5
Allergy	51 (1.4)	0.0–2.6	37 (1.0)	0.0–2.5
Immunodeficiency	9 (0.3)	0.0–0.9	8 (0.2)	0.0–0.9
Other	118 (3.3)	0.0–11.0	97 (2.7)	0.0–11.0
Other	1578 (44.1)	17.6–79.3	1371 (38.3)	17.6–76.4
Osteoporosis	485 (13.5)	0.0–23.2	438 (12.2)	0.0–22.0
Solid tumour (excluding C34)	304 (8.5)	2.6–25.2	261 (7.3)	0.0–24.4
Depression	287 (8.0)	0.0–31.3	251 (7.0)	0.0–30.5
Psoriasis	63 (1.8)	0.0–6.9	54 (1.5)	0.0–6.5

*C34* malignant neoplasm of bronchus and lung, *COPD* chronic obstructive pulmonary disease, *EMPIRE* European Multipartner IPF REgistry, *IPF* idiopathic pulmonary fibrosis

The frequency of comorbidities varied considerably between countries (Table 2; Additional file 1: Fig. S1). Data for each individual country are presented in Additional file 1: Figs. S1–7).

### Survival outcomes

Up to the end of 5 years of follow-up, 949 patients had died (Table 3). The most frequently reported cause of death was IPF-related respiratory failure (54.6%), followed by acute exacerbation of IPF (9.5%). Causes of death not considered IPF-related included cardiovascular disease (heart failure, cardiac arrest, stroke; 11.1%), pneumonia (4.9%), lung cancer (3.9%), other malignancy (2.7%) and other causes (3.5%). In total, comorbidity-related death was evidenced in at least 26.1% of cases, while cause of death was unknown in 9.8%.

**Table 3:** Causes of death in patients with IPF in the EMPIRE registry

Cause of death, n (%)	Patients (n = 939)	Antifibrotic treatment (n = 521)	No antifibrotic treatment (n = 418)
Progression of IPF; respiratory failure	513 (54.6)	274 (52.6)	239 (57.2)
Acute exacerbation of IPF	89 (9.5)	58 (11.1)	31 (7.4)
Heart failure	51 (5.4)	36 (6.9)	15 (3.6)
Pneumonia	46 (4.9)	22 (4.2)	24 (5.7)
Rhythmic disorder; cardiac arrest	44 (4.7)	25 (4.8)	19 (4.5)
Lung cancer	37 (3.9)	17 (3.3)	20 (4.8)
Other primary malignancy	25 (2.7)	15 (2.9)	10 (2.4)
Stroke	9 (1.0)	3 (0.6)	6 (1.4)
Other	33 (3.5)	20 (3.8)	13 (3.1)
Unknown	92 (9.8)	51 (9.8)	41 (9.8)

*EMPIRE* European Multipartner IPF REgistry, *IPF* idiopathic pulmonary fibrosis

Some associated disorders recorded at initial visit were associated with significantly increased risk of death in comparison with patients with no comorbidities (adjusted hazard ratio [HR]): namely pulmonary hypertension (1.93, *P* < 0.001), stroke (2.17, *P* < 0.001), respiratory infection (3.20, *P* = 0.031), emphysema (2.08, *P* < 0.001), lung cancer (3.39, *P* < 0.001), and obesity (1.92, *P* = 0.001). Patients with gastroesophageal reflux had slightly lower risk of death, with a borderline statistical significance (0.70, *P* = 0.043) (Table 4).

**Table 4:** Association between selected comorbidities (at enrolment) and mortality

Comorbidity	HR (95% CI)	*P*-value^a^	Adjusted HR (95% CI)^b^	*P*-value^a^
Cardiovascular	1.41 (1.08;1.844)	0.012	1.07 (0.794;1.437)	0.664
Arterial hypertension	1.29 (0.98;1.694)	0.068	0.96 (0.705;1.294)	0.766
Coronary heart disease	1.58 (1.18;2.116)	0.002	0.98 (0.695;1.387)	0.915
Pulmonary hypertension	2.88 (2.11;3.929)	< 0.001	1.93 (1.365;2.726)	< 0.001
Arrhythmias	1.50 (1.07;2.099)	0.017	1.16 (0.754;1.772)	0.506
Heart attack	1.69 (1.17;2.458)	0.005	1.19 (0.760;1.850)	0.453
Embolism	1.43 (0.94;2.174)	0.092	1.12 (0.683;1.851)	0.646
Stroke	1.43 (0.92;2.223)	0.113	2.17 (1.485;3.158)	< 0.001
Valve disease	1.40 (0.93;2.107)	0.104	0.87 (0.531;1.426)	0.581
Ischaemic disease of the lower limbs	1.01 (0.55;1.837)	0.985	0.70 (0.336;1.437)	0.326
Cardiomyopathy	0.95 (0.41;2.210)	0.903	0.42 (0.154;1.166)	0.096
Other	1.42 (1.00;1.999)	0.049	1.05 (0.681;1.604)	0.840
Pulmonary	1.84 (1.39;2.424)	< 0.001	1.26 (0.925;1.714)	0.142
Respiratory infection	4.95 (1.95;12.541)	0.001	3.20 (1.113;9.172)	0.031
COPD	1.33 (0.92;1.927)	0.125	0.92 (0.598;1.408)	0.695
Respiratory insufficiency	3.49 (2.36;5.146)	< 0.001	1.43 (0.901;2.267)	0.129
Asthma	0.94 (0.60;1.463)	0.771	1.05 (0.635;1.750)	0.839
Emphysema	2.54 (1.88;3.434)	< 0.001	2.08 (1.599;2.710)	< 0.001
Pneumonia	1.91 (0.94;3.860)	0.074	1.61 (0.918;2.815)	0.097
Lung cancer	5.04 (3.38;7.510)	< 0.001	3.39 (2.113;5.447)	< 0.001
Obstructive sleep apnoea	0.87 (0.45;1.663)	0.665	0.56 (0.278;1.133)	0.107
Tuberculosis	1.65 (1.19;2.285)	0.003	1.04 (0.717;1.517)	0.827
Other	3.40 (2.41;4.807)	< 0.001	1.85 (1.258;2.730)	0.002
Gastrointestinal/metabolic	1.28 (0.97;1.676)	0.080	1.01 (0.746;1.364)	0.957
Diabetes mellitus	1.29 (0.96;1.727)	0.095	0.93 (0.667;1.300)	0.674
Hyperlipidaemia	1.08 (0.80;1.452)	0.626	0.79 (0.562;1.101)	0.162
Gastroesophageal reflux	0.79 (0.58;1.087)	0.148	0.70 (0.490;0.989)	0.043
Obesity	1.45 (1.00;2.109)	0.050	1.92 (1.294;2.860)	0.001
Thyroid diseases	1.05 (0.72;1.530)	0.811	1.02 (0.631;1.656)	0.930
Hepatopathy	1.00 (0.62;1.574)	0.964	0.71 (0.419;1.210)	0.209
Other	1.37 (0.99;1.900)	0.058	0.91 (0.626;1.328)	0.629
Urogenital	1.19 (0.87;1.619)	0.270	0.82 (0.564;1.202)	0.314
Prostatic hypertrophy	1.18 (0.85;1.650)	0.321	0.76 (0.491;1.179)	0.222
Nephropathy	1.82 (1.17;2.809)	0.007	1.01 (0.596;1.720)	0.964
Other	1.06 (0.63;1.781)	0.837	0.75 (0.420;1.350)	0.341
Blood and immunity	1.65 (1.14;2.396)	0.008	1.35 (0.883;2.051)	0.168
Haematopoietic disorders	1.29 (0.75;2.206)	0.352	0.99 (0.509;1.925)	0.976
Allergy	1.59 (0.87;2.904)	0.135	1.12 (0.573;2.184)	0.742
Immunodeficiency	0.92 (0.13;6.696)	0.937	1.26 (0.171;9.298)	0.820
Other	1.67 (1.02;2.756)	0.043	1.66 (0.966;2.841)	0.067
Other disease	1.21 (0.92;1.603)	0.178	0.97 (0.711;1.333)	0.868
Osteoporosis	1.03 (0.74;1.440)	0.850	0.85 (0.571;1.272)	0.435
Solid tumor (exc. C34)	0.97 (0.68;1.395)	0.888	0.79 (0.511;1.236)	0.307
Depression	1.44 (0.98;2.118)	0.062	1.08 (0.708;1.643)	0.725
Psoriasis	0.86 (0.45;1.637)	0.642	0.72 (0.361;1.445)	0.358

*C34* malignant neoplasm of bronchus and lung, *CI* confidence interval, *COPD* chronic obstructive pulmonary disease, *FVC* forced vital capacity, *HR* hazard ratio, *NYHA* New York Heart Association functional classification

^a^*P* < 0.05 indicates significantly increased risk of death

^b^Adjusted for gender, age, smoking status and FVC% predicted at enrolment

**Fig. 1:**
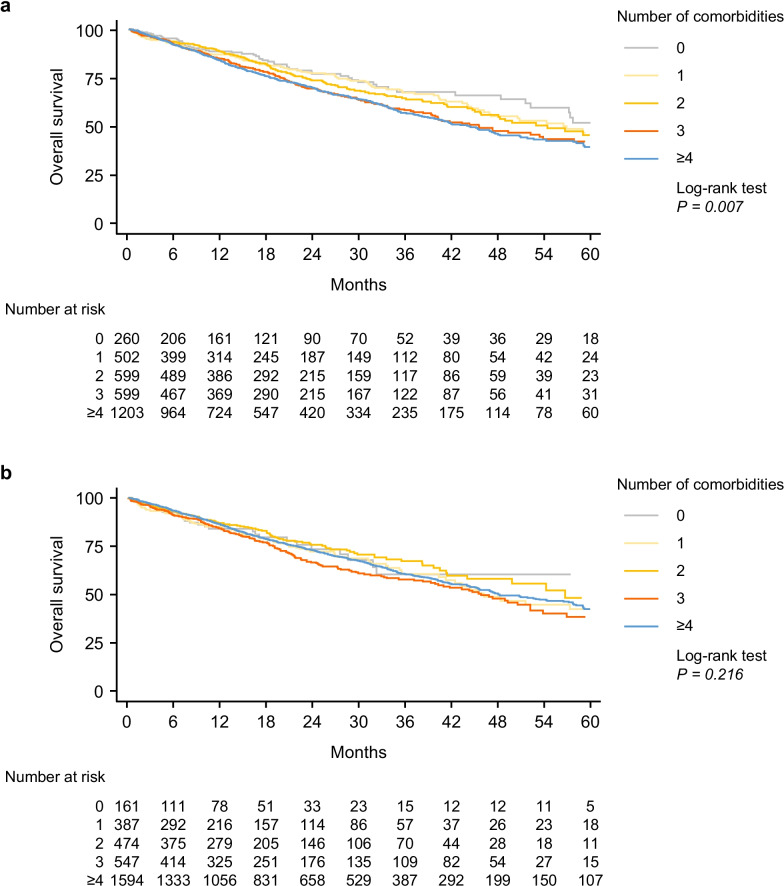
Overall survival in patients with IPF by number of comorbidities at enrolment and during follow-up. Comorbidities (**a**) prevalent at enrolment and (**b**) cumulative at enrolment and during follow-up (both n = 3580). IPF, idiopathic pulmonary fibrosis

The presence of multiple comorbidities at enrolment was associated with significantly worse survival (log-rank test *P* = 0.007) (Fig. 1A); and both Kaplan–Meier curves and Cox proportional hazards analysis indicated a dose–response-like relationship. In unadjusted Cox proportional hazards models, presence of ≥ 4 comorbidities at baseline compared with no comorbidity was associated with an unadjusted 51% increased risk of death (*P* = 0.004); following adjustment for gender, age, and FVC at baseline, the risk was 44% (*P* = 0.021).

Median overall survival was 52.1 months for the whole cohort. Median overall survival was not reached for those patients with no comorbidities at enrolment, whereas it was 58.9 months for those with 1 comorbidity, and 56.6, 47.2 and 45.5 months in patients with 2, 3 and ≥ 4 comorbidities, respectively (Fig. 1A). The 5-year overall probability of survival was 53.7% (95% confidence interval [CI] 40.8–64.9) in patients with no comorbidities, whereas it was 48.4% (39.3–56.9) in those with a 1 comorbidity, and 47.0% (38.4–55.2), 43.8% (36.6–50.9) and 41.1% (35.8–46.0) in patients with 2, 3 and ≥ 4 comorbidities respectively at enrolment.

The cumulative number of comorbidities (those recorded at enrolment and during follow-up combined) was not associated with survival (log-rank test *P* = 0.216); in addition, neither the unadjusted nor adjusted HR analysis indicated a dose–response-like relationship (Fig. 1B).

**Fig. 2:**
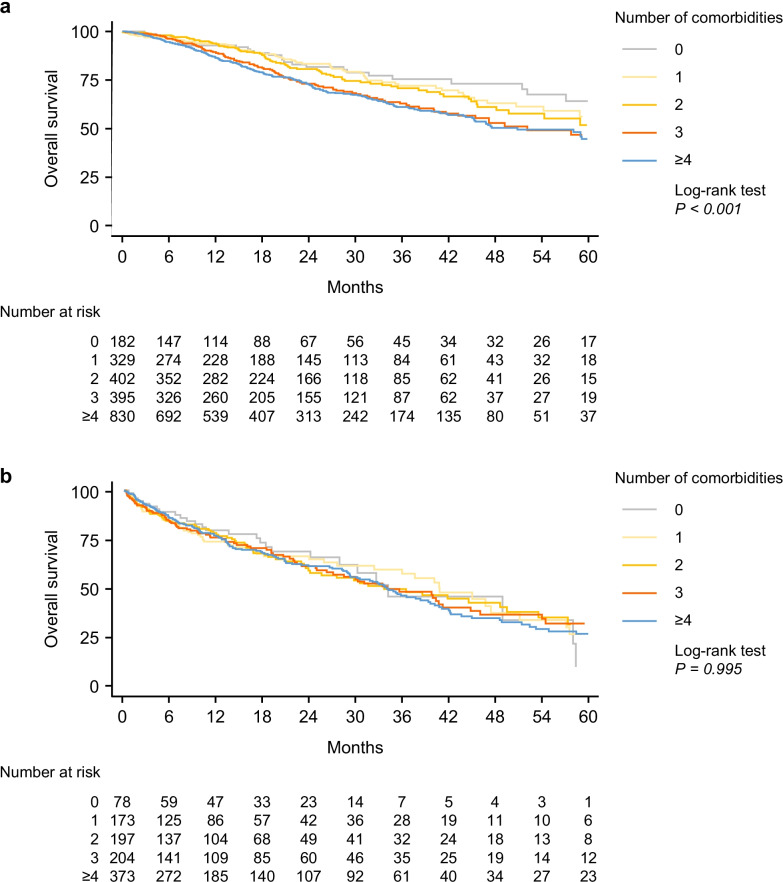
Overall survival in patients with IPF by comorbidities at enrolment according to antifibrotic therapy. Comorbidities for (**a**) those receiving antifibrotic therapy (n = 2257) and (**b**) those not receiving antifibrotic therapy (n = 1323). Antifibrotic therapies were nintedanib or pirfenidone. IPF, idiopathic pulmonary fibrosis

During 5 years of follow-up nearly two-thirds of patients (2,257/3,580; 63.0%) had received antifibrotic treatment with pirfenidone and/or nintedanib. Median (5th; 95th percentile) duration of antifibrotic treatment was 10.3 (0.9; 45.3) months (Additional file 1: Table S1). Median overall survival was 66.3 months for patients receiving antifibrotic treatment and 36.0 months for those not receiving antifibrotic treatment. Survival analysis of patients receiving antifibrotic treatment indicated a significantly different survival according to the number of comorbidities at enrolment (log-rank test *P* < 0.001) (Fig. 2A). Cox proportional hazards models also indicated increased risk of death in parallel with the number of comorbidities. In patients receiving antifibrotic treatment, median overall survival was not reached for those with none, 1, or 2 comorbidities, whereas it was 57.8 and 59.0 months for those with 3 or ≥ 4 comorbidities, respectively (Fig. 2A). In patients not receiving antifibrotic treatment, no association between survival and number of comorbidities was seen (log-rank test *P* = 0.995) (Fig. 2B).

## Discussion

### Main findings

The multinational EMPIRE registry includes patients with IPF managed in real-world settings across 11 countries of Central and Eastern Europe. In this analysis of 3,580 patients in the registry (2014–2020), the median number of comorbidities at enrolment was three, and a greater number of comorbidities was associated with significantly worse survival, resulting in a 41% increased risk of death for those with four or more comorbidities at baseline.

Analysis of patients receiving antifibrotic treatment indicated comparable differences in survival according to the number of comorbidities at enrolment (42% increased risk of death associated with four or more comorbidities).

### Comparison of reported comorbidities

Multimorbidity is known to be common in patients with IPF [12, 17, 21, 32] and, in agreement with published data, the majority of patients in the EMPIRE registry had at least one comorbidity. It is widely accepted that the risk of comorbidities such as arterial hypertension, coronary artery disease, lung cancer or osteoporosis increases with age, and this was confirmed in our study. Few studies provide detailed information on the number of comorbidities, and those have usually shown that multimorbidity is very common, especially in older IPF populations [12, 33], as reported in EMPIRE. Similarly, an analysis of 272 patients with IPF from a tertiary referral centre in Germany found that 89% had at least one comorbidity, and 30% had four or more comorbidities [9]. In another retrospective single-centre cohort of 352 patients with IPF, 79.3% had ≥ 1 comorbidities and 47.2% had ≥ 2 comorbidities [34].

The most frequently reported comorbidities in the EMPIRE registry were arterial hypertension, diabetes mellitus, hyperlipidaemia, coronary heart disease and, gastroesophageal reflux. Other registries have found similar patterns of comorbidities in IPF, but with sometimes strikingly different prevalence (Additional file 1: Fig. S8) [9, 16, 21, 35–38]. Publication of a systematic review of 126 clinical trials involving patients with IPF [12] has enabled comparisons to be made between highly scrutinised clinical trial populations and real-world patients from IPF registries [9, 16, 21, 35, 36]. The reported prevalence of comorbidities may differ, but almost all comorbidities listed are more common in clinical trial populations than in eight different registries, including EMPIRE (Additional file 1: Fig. S8) [9, 16, 21, 35–38]. This suggests that comorbidities are routinely and substantially under-recognised in clinical practice. Significant differences in reported comorbidities may be related to the type of study performed, which included retrospective analyses of single-centre experiences [9, 35], prospective or retrospective analyses of national registries [21, 36, 39] and analyses of administrative datasets [33, 40]. These differences are also dependent on the duration of the observation period [12, 41].

### Comorbidities and survival

Comorbidities are known to impact on IPF [23], and are linked to poor quality of life and increased mortality [42–45]. However, there is no clear answer on whether, and to what extent, comorbidities influence IPF progression in addition to their own direct adverse health effects.

In this EMPIRE analysis, the presence of multiple comorbidities at enrolment was associated with significantly worse survival. These findings are in line with published data [9].

However, it should be noted that a simple numerical increase in comorbidities does not necessarily result in worse outcomes, without considering the type and severity of the condition. In the present analysis, the cumulative number of comorbidities noted from enrolment up to the end of follow-up was not related to survival, possibly because comorbidities appearing later in the course of IPF may not have as big an impact on survival as those present earlier in the disease.

Previous reports have identified cardiovascular disease as a significant predictor of mortality in patients with IPF [9, 12, 46]; arteriosclerosis, other cardiovascular diseases (e.g., valvular heart disease), malignancy, and gastroesophageal reflux have all been associated with reduced survival in IPF [9, 12, 46]. Cardiovascular causes accounted for a substantial number of deaths in the EMPIRE population. Mortality has previously been reported to be higher among patients with IPF who had pulmonary comorbidities such as COPD, lung cancer and OSA [46, 47]. A number of comorbidities have therefore been found to be consistently associated with shorter survival in IPF [9, 12, 14, 46–49].

Recently, Torrisi et al. developed and validated the first-ever clinical prediction model and an index point score (TORVAN model and index) for all-cause mortality in IPF that includes comorbidities as parameters [23]. Relatively few comorbidities influenced the prediction of survival, and gender became a less important prognostic factor within the context of comorbidities [23].

### Antifibrotic treatment

Nearly two-thirds of all patients in this study had been prescribed pirfenidone or nintedanib, similarly to other studies [10, 16, 21, 36]. Treatment with antifibrotic therapy may indicate patients with more advanced disease stage or severity, which may be associated with more comorbidities. One of the main findings of this EMPIRE analysis was a significantly greater survival in patients with fewer comorbidities at enrolment. This finding was seen in patients treated with antifibrotics, but not in those who had not received antifibrotic treatment. Although there was no significant difference in the proportion of IPF-related versus non-IPF-related deaths between antifibrotic and non-antifibrotic-treated patients, the difference of survival as related to comorbidities was prominent only in those treated with antifibrotics, indicating that the influence of IPF itself on survival was lessened.

Recent analysis of the INSIGHT-IPF observational study demonstrated that antifibrotic treatment was associated with improved survival, independent from age [50], emphasising the importance of early introduction of antifibrotic treatment in IPF [50]—as also seen in the EMPIRE cohort.

### Study limitations

Over 3500 patients are included in the analyses and data were derived from 11 different countries; therefore, between-country differences in clinical practice, access to diagnostic procedures, availability of treatment for IPF, and healthcare system financing may have influenced the findings. Differences between countries in comorbidity frequency may be explained by inconsistent reporting, under-reporting, or differences in case definitions and applied diagnostic criteria. In addition, the earliest data were recorded before the publication of updated IPF treatment guidelines in 2015 [31], meaning that some patients may have received corticosteroids as well as antifibrotic therapy once it was available. Thus, reported longitudinal (survival) outcomes might reflect different treatment approaches over time. Another limitation is that the analysis of survival by number of comorbidities does not account for differences in type or severity of comorbidity. However, the survival analysis was adjusted for gender, age, smoking status and FVC% predicted at baseline as these factors could be associated with comorbidities and may influence survival [11, 12].

## Summary

Patient registries can provide valuable real-world information about the prevalence of comorbidities. Findings from the EMPIRE registry in Central and Eastern Europe, the largest IPF registry in the world, indicate that multimorbidity is common. The presence of multiple comorbidities at enrolment appears to be associated with worse survival, an effect particularly present in patients receiving antifibrotic treatment.

In conclusion, comorbidities in IPF are common but may often be under-recognised in clinical practice. Raising awareness of the impact that comorbidities can have on these patients would help to improve recognition and management in clinical practice. Optimising detection and management of comorbidities would help to improve outcomes in patients with IPF; with improved survival associated with antifibrotic treatment, the influence of comorbidities now has greater influence than previously.

## Supplementary Information

**Additional file 1: Table S1.** Duration of antifibrotic therapies. **Table S2.** Survival of patients with IPF by number of comorbidities. **Table S3.** Survival of patients with IPF by number of comorbidities according to antifibrotic therapy. **Figure S1.** Comorbidities by body system in participating EMPIRE countries. **Figure S2.** Cardiovascular comorbidities in participating EMPIRE countries. **Figure S3.** Metabolic and gastrointestinal comorbidities in participating EMPIRE countries. **Figure S4.** Pulmonary comorbidities in participating EMPIRE countries. **Figure S5.** Urogenital comorbidities in participating EMPIRE countries. **Figure S6.** Blood and immunity disorders in participating EMPIRE countries. **Figure S7.** Other comorbidities in participating EMPIRE countries. **Figure S8.** Comorbidities in patients with IPF in EMPIRE compared other real-world registries and clinical trials.

## Data Availability

Data can be requested from the EMPIRE registry following approval from the steering committee.

## Abbreviations

CI: Confidence interval
COPD: Chronic obstructive pulmonary disease
EMPIRE: European MultiPartner IPF REgistry
FVC: Forced vital capacity
HR: Hazard ratio
IPF: Idiopathic pulmonary fibrosis
MDT: Multidisciplinary team
OSA: Obstructive sleep apnoea
