# Supplementary material for: Comorbidity burden and survival in patients with idiopathic pulmonary fibrosis: the EMPIRE registry study
Source: Respir Res. 2022 May 27;23:135. doi: 10.1186/s12931-022-02033-6 (PMC9145164; doi:10.1186/s12931-022-02033-6)
Supplement: Supplementary file 1 — Additional file 1: Table S1. Duration of antifibrotic therapies. Table S2. Survival of patients with IPF by number of comorbidities. Table S3. Survival of patients with IPF by number of comorbidities according to antifibrotic therapy. Figure S1. Comorbidities by body system in participating EMPIRE countries. Figure S2. Cardiovascular comorbidities in participating EMPIRE countries. Figure S3. Metabolic and gastrointestinal comorbidities in participating EMPIRE countries. Figure S4. Pulmonary comorbidities in participating EMPIRE countries. Figure S5. Urogenital comorbidities in participating EMPIRE countries. Figure S6. Blood and immunity disorders in participating EMPIRE countries. Figure S7. Other comorbidities in participating EMPIRE countries. Figure S8. Comorbidities in patients with IPF in EMPIRE compared other real-world registries and clinical trials. [file 12931_2022_2033_MOESM1_ESM.docx]

**Additional file 1**

**Supplementary Table S1.** **Duration of antifibrotic therapies**

|  | **Total** | **Pirfenidone** | **Nintedanib** | **Switch pirfenidone  to nintedanib** | **Switch nintedanib to pirfenidone** |
| --- | --- | --- | --- | --- | --- |
|  | **(n = 841)** | **(n = 418)** | **(n = 181)** | **(n = 165)** | **(n = 77)** |
| **Mean duration of therapy, months (SD)** | 15.0 (14.8) | 16.1 (15.7) | 15.6 (14.4) | 12.8 (13.4) | 12.7 (13.5) |
| **Median duration of therapy, months (5; 95 percentile)** | 10.3 (0.9; 45.2) | 11.0 (0.8; 48.6) | 11.3 (0.9; 42.7) | 7.5 (1.0; 43.1) | 7.5 (0.6; 33.3) |

SD, standard deviation.

**Supplementary Table S2. Survival of patients with IPF by number of comorbidities**

| **Number of comorbidities** | **Median survival, months** | **1-year survival  (95% CI)** | **3-year survival**  **(95% CI)** | **5-year survival  (95% CI)** | **Significant differences between number of comorbidities^1^** |
| --- | --- | --- | --- | --- | --- |
| **At enrolment** | | | | | |
| 0 | - | 88.9  (83.9; 92.5) | 69.2  (60.2; 76.6) | 53.7  (40.8; 64.9) | 3, ≥ 4 |
| 1 | 58.9 | 87.5  (84.1; 90.3) | 68.4  (62.4; 73.6) | 48.4  (39.3; 56.9) | ≥ 4 |
| 2 | 56.6 | 88.7  (85.7; 91.2) | 64.9  (59.3; 69.9) | 47.0  (38.4; 55.2) | ≥ 4 |
| 3 | 47.2 | 85.3  (81.9; 88.1) | 59.5  (54.0; 64.6) | 43.8  (36.6; 50.9) | 0 |
| ≥ 4 | 45.5 | 83.9  (81.5; 86.0) | 57.9  (54.0; 61.6) | 41.1  (35.8; 46.3) | 0, 1, 2 |
| **During follow-up^2^** | | | | | |
| 0 | - | 84.5  (76.6; 89.9) | 61.5  (46.0; 73.8) | 61.5  (46.0; 73.8) | - |
| 1 | 48.3 | 85.8  (81.5; 89.2) | 61.9  (53.9; 68.9) | 44.4  (33.4; 54.7) | - |
| 2 | 56.6 | 87.3  (83.6; 90.3) | 68.2  (61.5; 74.0) | 49.7  (37.0; 61.3) | - |
| 3 | 47.2 | 84.7  (81.0; 87.7) | 59.4  (53.6; 64.7) | 40.5  (32.2; 48.7) | - |
| ≥ 4 | 51.7 | 86.4  (84.5; 88.1) | 61.5  (58.4; 64.6) | 44.1  (39.8; 48.3) | - |

CI, confidence interval; IPF, idiopathic pulmonary fibrosis.

^1^Multiple comparisons using Bonferroni correction.

^2^Accumulative number of comorbidities from enrolment and during follow-up.

**Supplementary Table S3. Survival of patients with IPF by number of comorbidities according to antifibrotic therapy^1^**

| **Number of comorbidities** | **Median survival, months** | **1-year survival  (95% CI)** | **3-year survival**  **(95% CI)** | **5-year survival  (95% CI)** | **Significant differences between number of comorbidities^2^** |
| --- | --- | --- | --- | --- | --- |
| **With antifibrotic therapy** | | | | | |
| 0 | - | 93.2  (87.6; 96.3) | 77.6  (67.5; 85.0) | 66.8  (52.7; 77.5) | 3, ≥ 4 |
| 1 | - | 93.9  (90.4; 96.1) | 73.3  (66.0; 79.3) | 58.3  (47.2; 67.8) | 3, ≥ 4 |
| 2 | - | 94.2  (91.1; 96.2) | 72.1  (65.4; 77.7) | 54.2  (42.7; 64.3) | 4 |
| 3 | 57.8 | 89.9  (86.0; 92.7) | 64.6  (57.9; 70.6) | 49.3  (39.7; 58.2) | 0, 1 |
| ≥ 4 | 59.0 | 87.0  (84.3; 89.3) | 63.0  (58.3; 67.2) | 47.2  (40.3; 53.8) | 0, 1, 2 |
| **No antifibrotic therapy** | | | | | |
| 0 | 33.7 | 80.0  (68.4; 87.7) | 46.9  (27.5; 64.2) | 11.7  (0.8; 38.7) | - |
| 1 | 40.2 | 74.9  (66.9; 81.2) | 58.6  (47.7; 68.1) | 28.3  (15.0; 43.1) | - |
| 2 | 36.0 | 77.1  (69.9; 82.8) | 49.2  (39.0; 58.6) | 32.8  (20.9; 45.3) | - |
| 3 | 35.1 | 76.4  (69.3; 82.1) | 49.3  (39.7; 58.2) | 33.4  (22.9; 44.3) | - |
| ≥ 4 | 34.3 | 76.7  (71.6; 81.1) | 46.6  (39.4; 53.5) | 28.4  (20.8; 36.4) | - |

CI, confidence interval; IPF, idiopathic pulmonary fibrosis.

^1^Nintedaninb or pirfenidone.

^2^Multiple comparisons using Bonferroni correction.

**Supplementary Figure S1. Comorbidities by body system in participating EMPIRE countries**


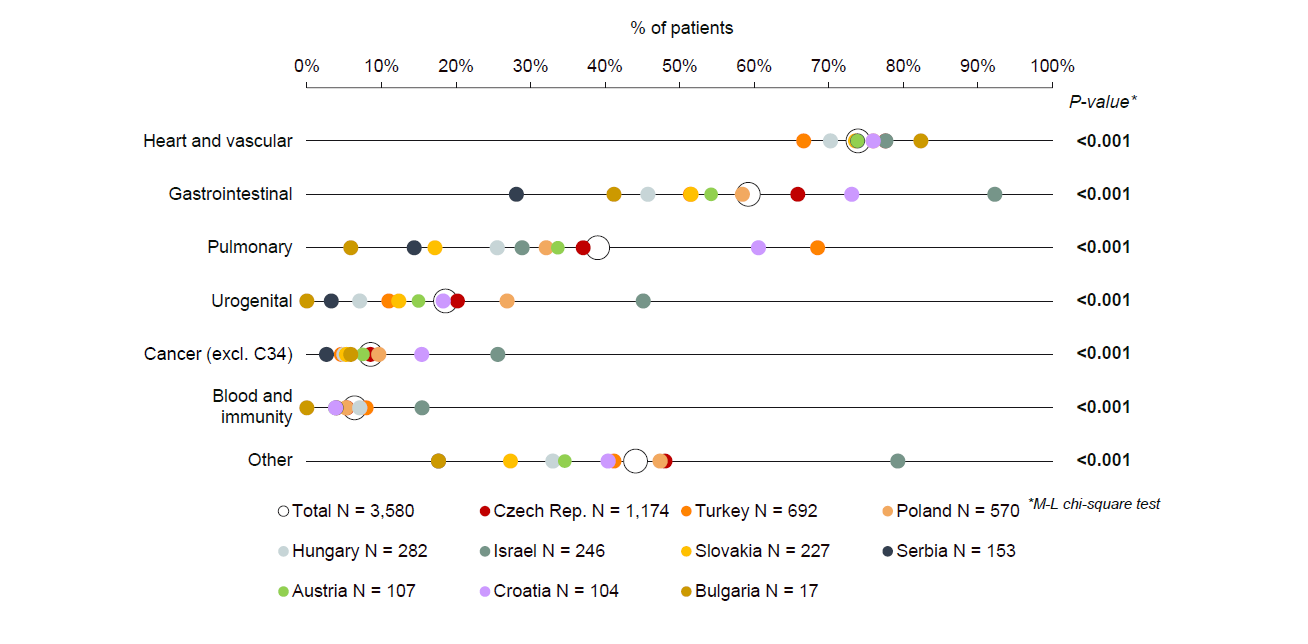


C34, malignant neoplasm of bronchus and lung; EMPIRE, European MultiPartner IPF REgistry; ML, maximum likelihood**.**

**Supplementary Figure S2. Cardiovascular comorbidities in participating EMPIRE countries**


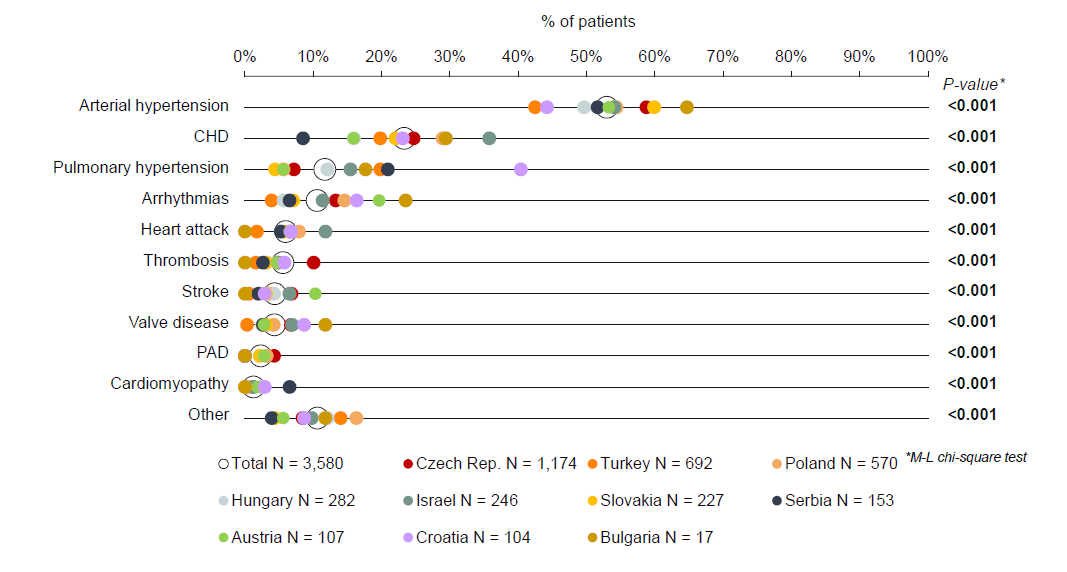


EMPIRE, European MultiPartner IPF REgistry; ML, maximum likelihood.

**Supplementary Figure S3. Metabolic and gastrointestinal comorbidities in participating EMPIRE countries**


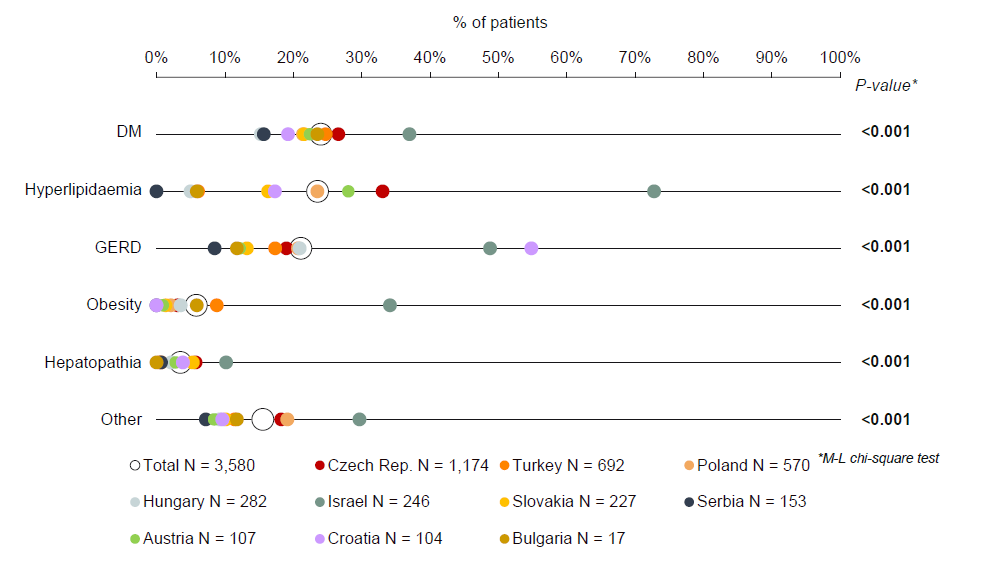


EMPIRE, European MultiPartner IPF REgistry; GERD, gastroesophageal reflux disease; ML, maximum likelihood**.**

**Supplementary Figure S4. Pulmonary comorbidities in participating EMPIRE countries**


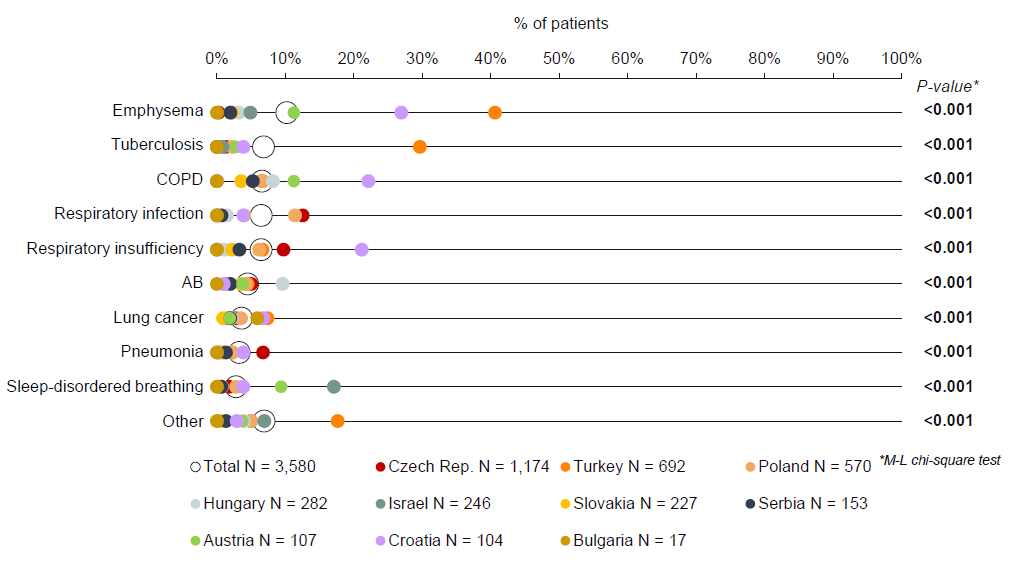


COPD, chronic obstructive pulmonary disease; EMPIRE, European MultiPartner IPF REgistry; ML, maximum likelihood.

**Supplementary Figure S5. Urogenital comorbidities in participating EMPIRE countries**


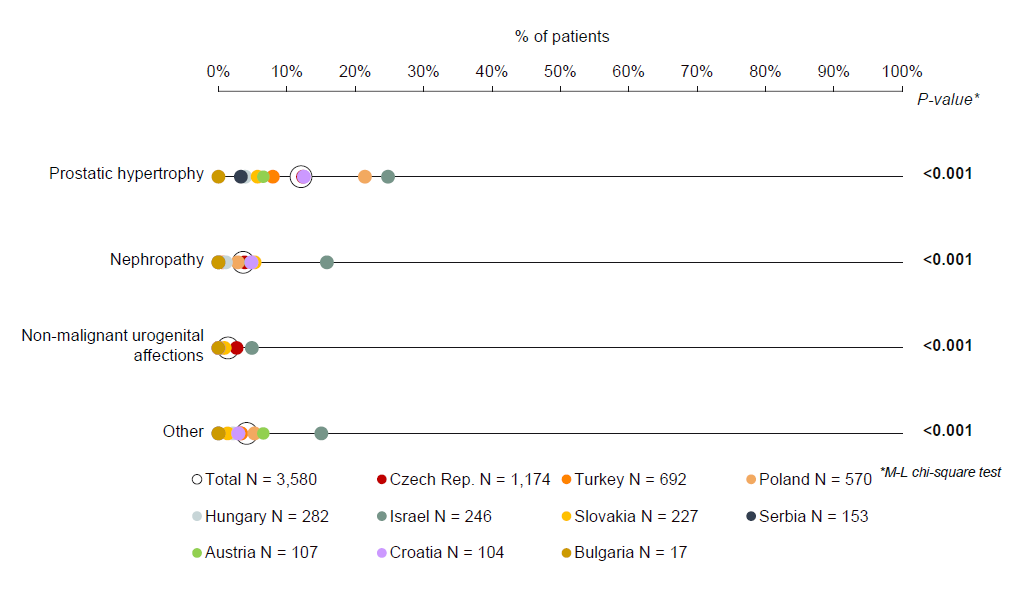


EMPIRE, European MultiPartner IPF REgistry; ML, maximum likelihood.

**Supplementary Figure S6. Blood and immunity disorders in participating EMPIRE countries**


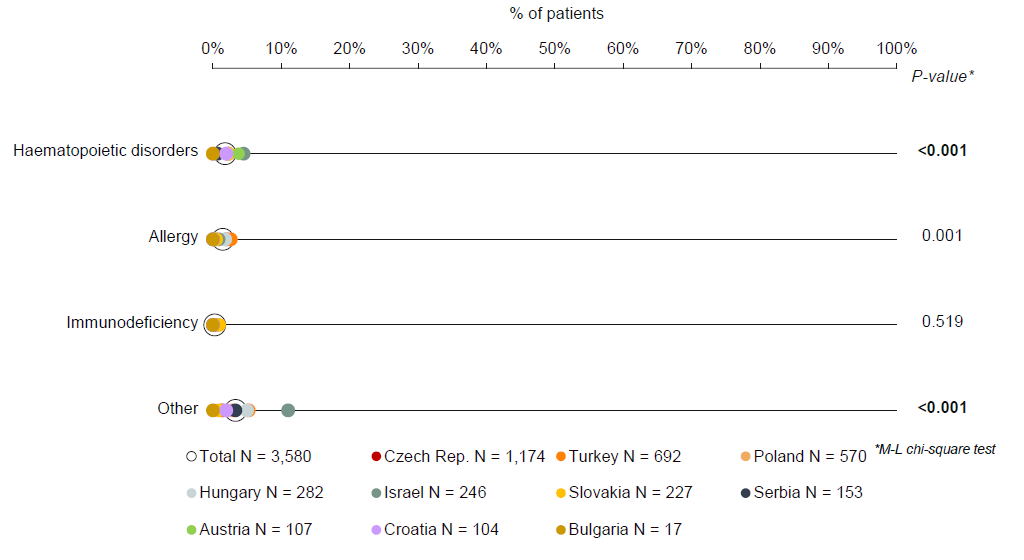


EMPIRE, European MultiPartner IPF REgistry; ML, maximum likelihood.

**Supplementary Figure S7. Other comorbidities in participating EMPIRE countries**


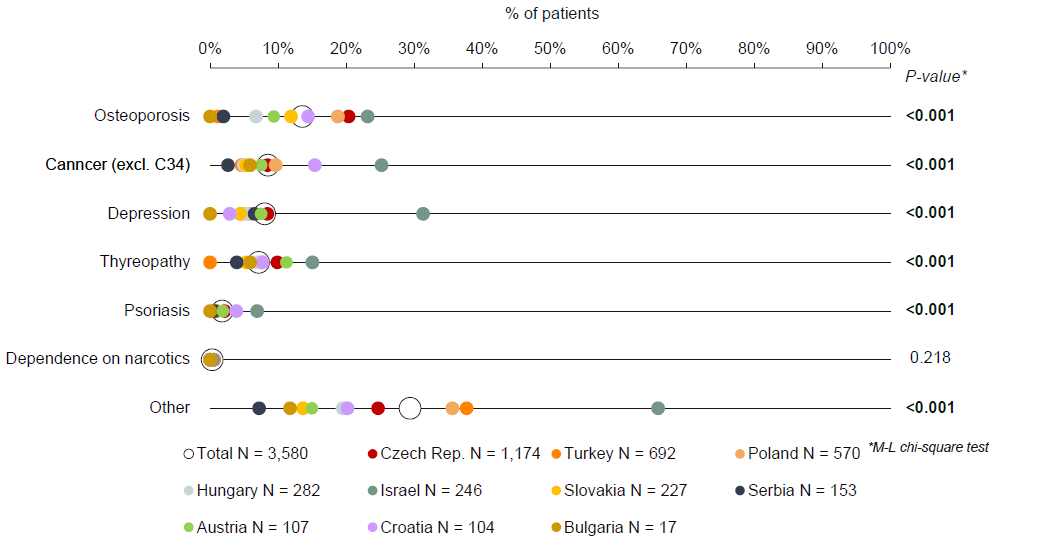


C34, malignant neoplasm of bronchus and lung; EMPIRE, European MultiPartner IPF REgistry; ML, maximum likelihood.

**Supplementary Figure S8. Comorbidities in patients with IPF in EMPIRE compared other real-world registries and clinical trials**

Data on comorbidities (a and b) are summarised from EMPIRE, eight other real-world registries or cohorts (PROOF registry [1], INSIGHTS-IPF registry [2], European IPF registry [3], IPF-PRO registry [4], FIBRONET registry [5], the PASSPORT registry [6], Danish database [7], German database [8]), as well as a systematic review of 126 clinical trials [9].


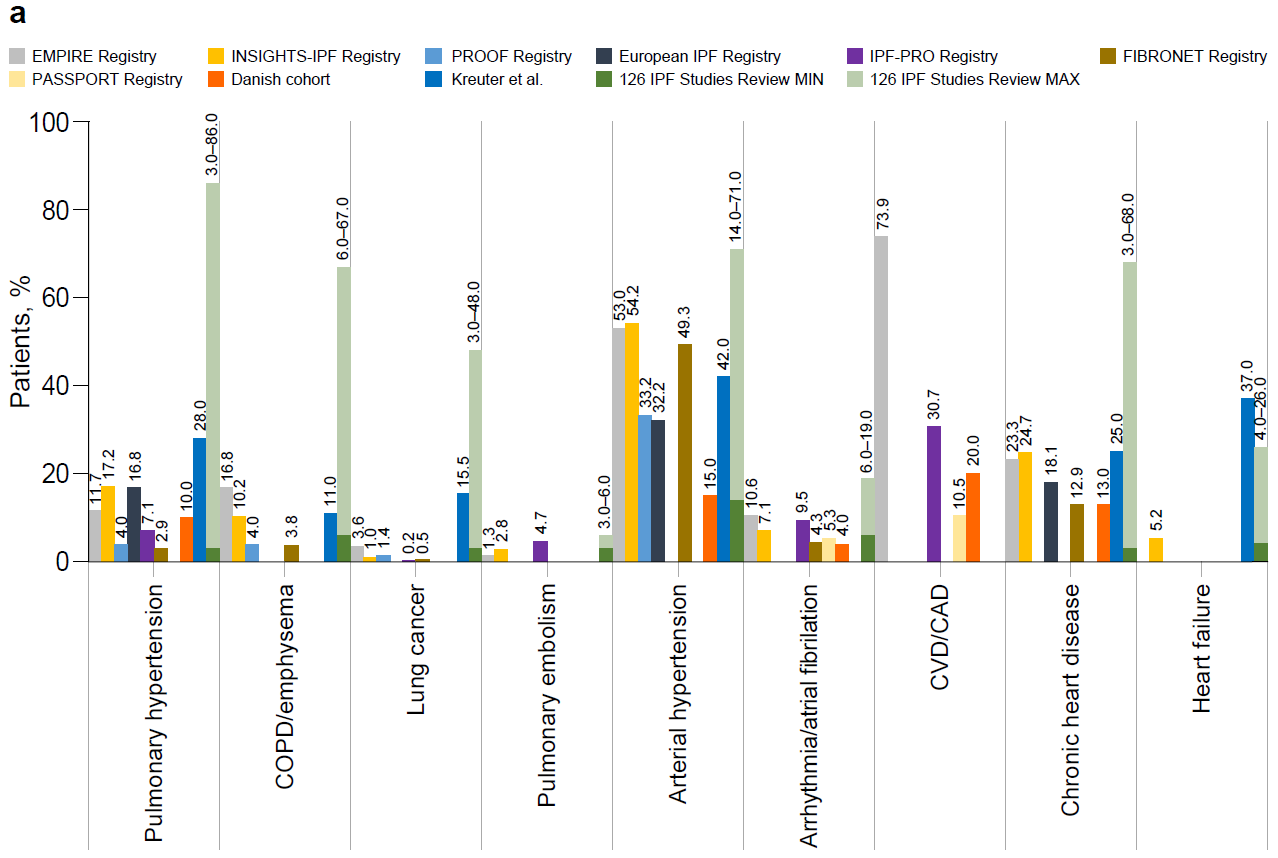


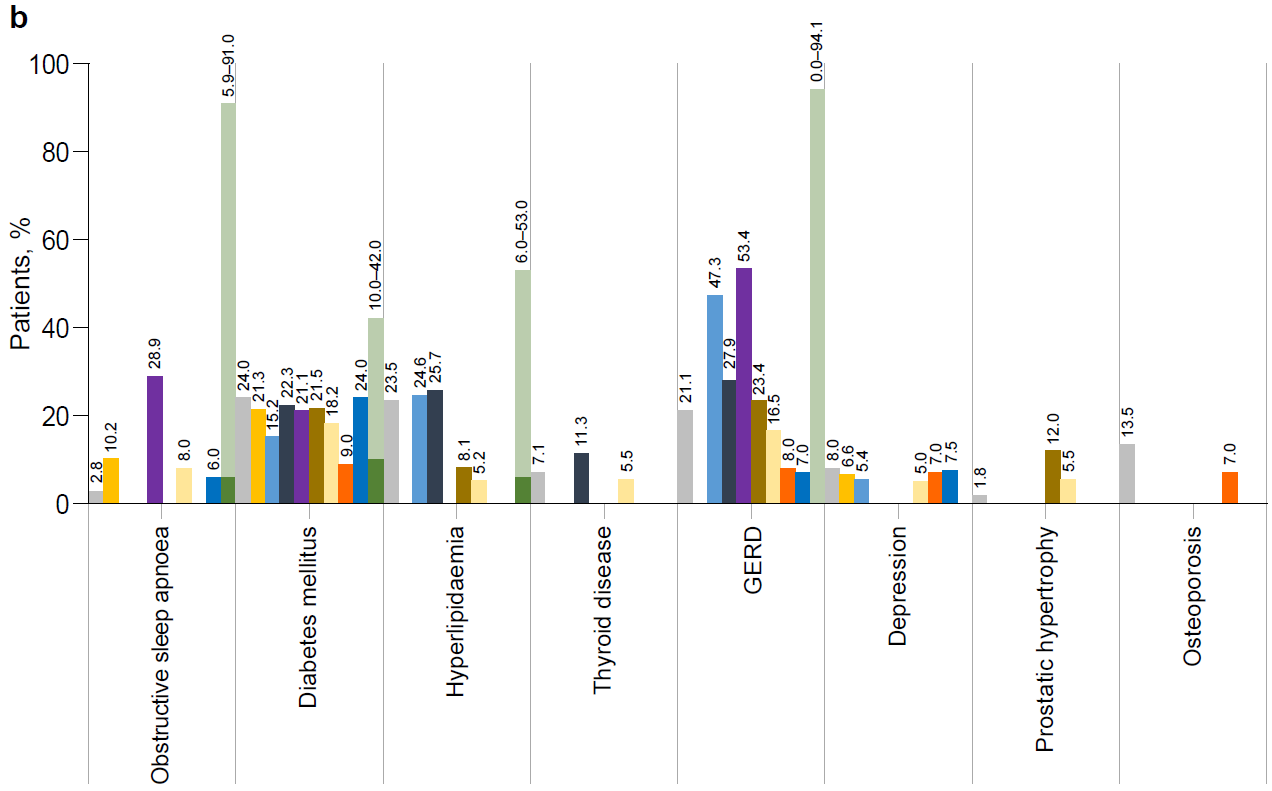


CAD, coronary artery disease; CHD, chronic heart disease; COPD, chronic obstructive pulmonary disease; CVD, cardiovascular disease; EMPIRE, European MultiPartner IPF REgistry; GERD, gastroesophageal reflux disease; IPF, idiopathic pulmonary fibrosis.

**References**

1. Wuyts WA, Dahlqvist C, Slabbynck H, Schlesser M, Gusbin N, Compere C, et al. Baseline clinical characteristics, comorbidities and prescribed medication in a real-world population of patients with idiopathic pulmonary fibrosis: the PROOF registry. BMJ Open Respir Res. 2018;5:e000331.

2. Behr J, Kreuter M, Hoeper MM, Wirtz H, Klotsche J, Koschel D, et al. Management of patients with idiopathic pulmonary fibrosis in clinical practice: the INSIGHTS-IPF registry. Eur Respir J. 2015;46:186-96.

3. Guenther A, Krauss E, Tello S, Wagner J, Paul B, Kuhn S, et al. The European IPF registry (eurIPFreg): baseline characteristics and survival of patients with idiopathic pulmonary fibrosis. Respir Res. 2018;19:141.

4. Snyder LD, Mosher C, Holtze CH, Lancaster LH, Flaherty KR, Noth I, et al. Time to diagnosis of idiopathic pulmonary fibrosis in the IPF-PRO Registry. BMJ Open Respir Res. 2020;7.

5. Poletti V, Vancheri C, Albera C, Harari S, Pesci A, Metella RR, et al. Clinical course of IPF in Italian patients during 12 months of observation: results from the FIBRONET observational study. Respir Res. 2021;22:66.

6. Cottin V, Koschel D, Gunther A, Albera C, Azuma A, Skold CM, et al. Long-term safety of pirfenidone: results of the prospective, observational PASSPORT study. ERJ Open Res. 2018;4.

7. Hyldgaard C, Hilberg O, Bendstrup E. How does comorbidity influence survival in idiopathic pulmonary fibrosis? Respir Med. 2014;108:647-53.

8. Kreuter M, Ehlers-Tenenbaum S, Palmowski K, Bruhwyler J, Oltmanns U, Muley T, et al. Impact of Comorbidities on Mortality in Patients with Idiopathic Pulmonary Fibrosis. PLoS One. 2016;11:e0151425.

9. Raghu G, Amatto VC, Behr J, Stowasser S. Comorbidities in idiopathic pulmonary fibrosis patients: a systematic literature review. Eur Respir J. 2015;46:1113-30.
